# Sinking Skin Flap Syndrome, a Rare Complication of Craniectomy

**DOI:** 10.5334/jbsr.2821

**Published:** 2022-05-27

**Authors:** Martin Cassagne, Anne-Sophie Claes

**Affiliations:** 1 Hôpital de Jolimont, BE

**Keywords:** Sinking skin flap syndrome, paradoxical herniation, syndrome of the trephined, craniectomy

## Abstract

**Teaching point:** Sinking skin flap syndrome is a medical emergency that rarely complicates large craniectomy. It results from an intracerebral hypotension and requires the replacement of the cranial flap.

## Case

**Figure 1:**
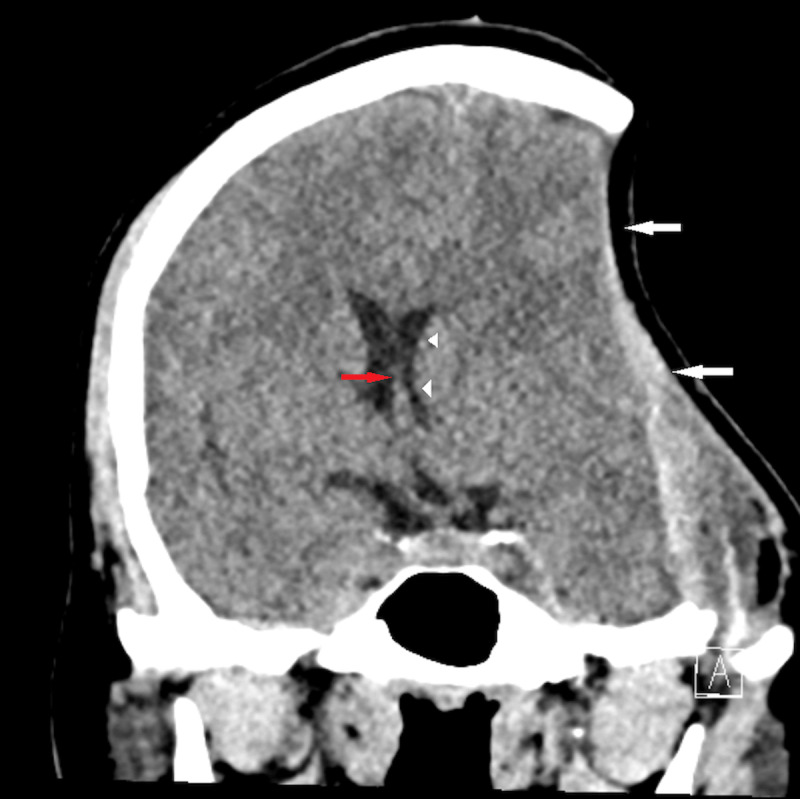


**Figure 2:**
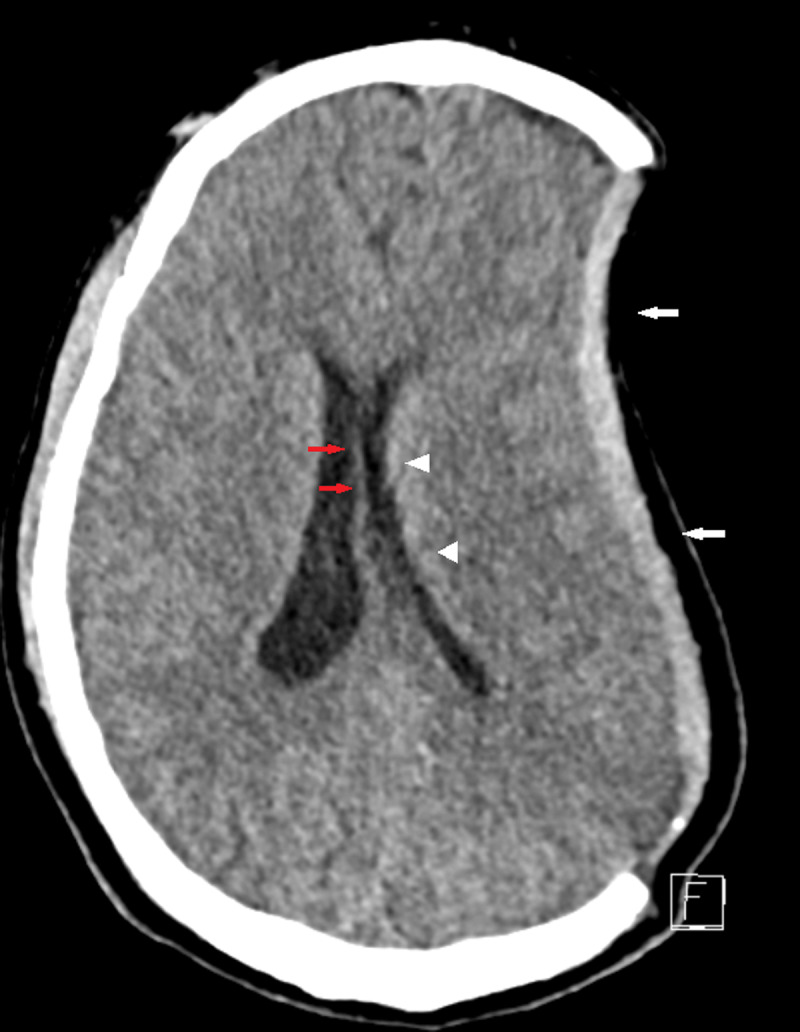


A 23-year-old man was referred to the emergency department after a fall and neurological deterioration. Two months earlier, he underwent a large left decompressive craniectomy after major traumatic brain injury. The cranial CT showed a large concavity of the overlying skin flap (white arrow, Figures 1 and 2), and cerebral mass effect with less visibility of the sulci, partially compressed left lateral ventricle (arrowhead, Figures 1 and 2) and subfalcine right brain herniation (red arrow, Figures 1 and 2). The combination of those signs is evocative of the sinking skin flap syndrome, also known as syndrome of the trephined or paradoxal hernia.

## Discussion

The sinking skin flap syndrome (SSFS) or syndrome of the trephined is a rare complication that occurs in approximately 10% of large craniectomies and tends to develop several weeks to several months after surgery. It consists of a sunken scalp above the bone defect with neurological symptoms.

The principal symptoms are severe orthostatic headache, motor deficits, cognitive decline or seizures. The SSFS may progress to “paradoxal hernia” and eventually lead to coma or death without treatment.

Several hypothesis have been proposed to explain the physiopathology of this syndrome which nevertheless remains unclear. One theory suggests a direct compression of the brain by the atmospheric pressure to the intracranial cavity through the skin scalp. Another hypothesis proposed that the difference between atmospheric and intracranial pressure may lead to hypovolemia and/or hypopressure in the cerebrospinal fluid (CSF). CSF drainage such as external ventriculostomies, ventriculoperitoneal shunts, or after lumbar punctures can aggravated this condition. Some authors suggest that craniectomy may induce significant alterations in blood flow regulation mechanisms. All these hypothesis may contribute to decreased regional cerebral blood flow and metabolic changes causing cortical dysfunction and neurological deficits [1].

CT scan revealed a large craniectomy with concavity of the overlying skin scalp, with mass effect such as sulci effacement and midline shift in the opposite direction of the scalp, also referred to as ‘paradoxal hernia.’

The final treatment is cranioplasty with replacement of the cranial flap but surgery is often delayed for many reasons (infections, …). During this time, measures are needed to raise the intracranial pressure like Trendelenburg position, hydration and clamping of CSF drainage.
